# Implementation strategies and outcomes for occupational therapy in adult stroke rehabilitation: a scoping review

**DOI:** 10.1186/s13012-021-01178-0

**Published:** 2021-12-18

**Authors:** J. Edward Murrell, Janell L. Pisegna, Lisa A. Juckett

**Affiliations:** 1 Department of Health Services Administration, School of Health Professions, The University of Alabama at Birmingham, Birmingham, AL USA; 2 Division of Occupational Therapy, School of Health and Rehabilitation Sciences, The Ohio State University, Columbus, OH USA

**Keywords:** Implementation strategies, Implementation outcomes, Occupational therapy, Stroke rehabilitation

## Abstract

**Background:**

Stroke survivors often encounter occupational therapy practitioners in rehabilitation practice settings. Occupational therapy researchers have recently begun to examine the implementation strategies that promote the use of evidence-based occupational therapy practices in stroke rehabilitation; however, the heterogeneity in how occupational therapy research is reported has led to confusion about the types of implementation strategies used in occupational therapy and their association with implementation outcomes. This review presents these strategies and corresponding outcomes using uniform language and identifies the extent to which strategy selection has been guided by theories, models, and frameworks (TMFs).

**Methods:**

A scoping review protocol was developed to assess the breadth and depth of occupational therapy literature examining implementation strategies, outcomes, and TMFs in the stroke rehabilitation field. Five electronic databases and two peer-reviewed implementation science journals were searched to identify studies meeting the inclusion criteria. Two reviewers applied the inclusion parameters and consulted with a third reviewer to achieve consensus. The 73-item Expert Recommendations for Implementing Change (ERIC) implementation strategy taxonomy guided the synthesis of implementation strategies. The Implementation Outcomes Framework guided the analysis of measured outcomes.

**Results:**

The initial search yielded 1219 studies, and 26 were included in the final review. A total of 48 out of 73 discrete implementation strategies were described in the included studies. The most used implementation strategies were “distribute educational materials” (*n* = 11), “assess for readiness and identify barriers and facilitators” (*n* = 11), and “conduct educational outreach visits” (*n* = 10). “Adoption” was the most frequently measured implementation outcome, while “cost” was not measured in any included studies. Eleven studies reported findings supporting the effectiveness of their implementation strategy or strategies; eleven reported inconclusive findings, and four found that their strategies did not lead to improved implementation outcomes. In twelve studies, at least partially beneficial outcomes were reported, corresponding with researchers using TMFs to guide implementation strategies.

**Conclusions:**

This scoping review synthesized implementation strategies and outcomes that have been examined in occupational therapy and stroke rehabilitation. With the growth of the stroke survivor population, the occupational therapy profession must identify effective strategies that promote the use of evidence-based practices in routine stroke care and describe those strategies, as well as associated outcomes, using uniform nomenclature. Doing so could advance the occupational therapy field’s ability to draw conclusions about effective implementation strategies across diverse practice settings.

**Supplementary Information:**

The online version contains supplementary material available at 10.1186/s13012-021-01178-0.

Contributions to the literature

- This review advances the occupational therapy profession’s awareness of the implementation strategies that have been applied and evaluated in stroke rehabilitation.
- Consistent use of implementation science theories, models, and frameworks, such as the Expert Recommendations for Implementation Change (ERIC) project and the Implementation Outcomes Framework (IOF), can elucidate occupational therapy researchers’ understanding of implementation strategies that lead to improved implementation outcomes.
- It remains unclear which implementation strategies are most effective for improving implementation outcomes in the context of stroke rehabilitation and occupational therapy. Notably, only 12 of 26 studies included in this review were guided by an implementation theory, model, or framework, which may partially explain the variability in study findings.

## Background

Every year, millions of people worldwide experience a stroke [1, 2]. In 2016 alone, there were over 13 million new cases of stroke globally [3]. At elevated risk for stroke are persons who are 65 and older, practice unhealthy behaviors (smoking, poor diet, and physical inactivity), have metabolic risks (high blood pressure, high glucose, decreased kidney function, obesity, and high cholesterol), and represent lower socioeconomic groups [1, 4, 5]. With the rapid growth of the older adult population, the number of stroke survivors is expected to rise dramatically in the coming years, contributing to a shift in increased global disease burden [6–9]. Stroke is one of the leading causes of long-term disability worldwide, and stroke survivors often face extensive challenges that result in self-care dependency, mobility impairments, underemployment, and cognitive deficits [1, 10]. Frequently, stroke survivors are admitted to stroke rehabilitation settings, such as outpatient care centers, skilled nursing facilities, and home health agencies. Occupational therapy (OT) practitioners work with stroke survivors in these settings to address their physical, cognitive, and psychosocial challenges [10–13]. Considered allied health professionals, OT practitioners across the stroke rehabilitation continuum are expected to implement a person-centered care plan using evidence-based assessments and interventions intended to maximize stroke survivors’ independence in daily activities and routines (e.g., dressing, bathing, mobility). Furthermore, healthcare users (e.g., stroke survivors) expect practitioners to deliver evidence-based practice and provide the highest quality occupational therapy services.

The benefits of OT in stroke rehabilitation have been well documented [14]. For instance, evidence-based OT interventions can lead to improved upper extremity movement [15, 16], enhanced cognitive performance [17], and increased safety with mobility [18]. However, as with several allied health professions, OT practitioners can experience complex barriers when implementing evidence-based care into routine practice [19–21]. Specific to stroke rehabilitation, Juckett et al. [22] identified several barriers that limited OT practitioners’ use of evidence and categorized these barriers according to the Consolidated Framework for Implementation Research (CFIR) [23]. Notable barriers to evidence use were attributed to challenges adapting evidence-based programs and interventions to meet patients’ needs (e.g., adaptability), a lack of equipment and personnel (e.g., available resources), and insufficient internal communication systems (e.g., networks and communication). Although identifying these barriers is a necessary precursor to optimizing evidence implementation, Juckett et al. [22] also emphasized the urgent need for OT researchers and practitioners to identify *implementation strategies* that facilitate the use of evidence in stroke rehabilitation. Relatedly, Jones et al. [24] examined the literature regarding implementation strategies used in the rehabilitation profession: occupational therapy, physical therapy, and speech–language pathology. While they found some encouraging findings, it is difficult to replicate these strategies given the heterogeneity in how implementation strategies and outcomes were defined and the inconsistency with which implementation strategy selection was informed by implementation theories, models, and frameworks (TMFs) [24]. Just as it is critical to select implementation strategies based on known implementation barriers, the design of implementation studies should be guided by TMFs to optimize the generalizability of findings towards both implementation and patient outcomes [25].

Implementation strategies are broadly defined as methods to enhance the adoption, use, and sustainment of evidence-based interventions, programs, or innovations [26, 27]. Historically, the terminology and definitions used to describe implementation strategies have been inconsistent and lacking details [28–30]. Over the past decade, however, these strategies have been compiled into taxonomies and frameworks to facilitate researchers’ and practitioners’ ability to conceptualize, apply, test, and describe implementation strategies utilized in research and practice. The Expert Recommendations for Implementing Change (ERIC) project [28] describes a taxonomy of 73 discrete implementation strategies that have been leveraged to optimize the use of evidence in routine care [29, 31]. Additionally, as part of the ERIC project, an expert panel examined the relationships among the discrete implementation strategies to determine any themes and to categorize strategies in clusters [29]. Table 1 depicts how discrete implementation strategies are organized in the following clusters: *use evaluative and iterative strategies, provide interactive assistance, adapt and tailor to the context, develop stakeholder interrelationships, train and educate stakeholders, support clinicians, engage consumers, utilize financial strategies, and change infrastructure*.

**Table 1:** Summary of implementation strategies utilized in terms of ERIC^a^ thematic clusters [29]^b^

		Studies (***N*** = 26)		Implementation strategies (***N*** = 150)		Discrete IS^c^ within cluster			
	ERIC thematic cluster	***n***	%	***n***	%	***N***	***n***	%	ERIC taxonomy of implementation strategies^d^
1	Use evaluative and iterative strategies	17	65	31	21	10	9	90	Assess for readiness and identify barriers and facilitators | Audit and provide feedback | Conduct cyclical small tests of change | Develop a formal implementation blueprint | Develop and implement tools for quality monitoring | Develop and organize quality monitoring systems | Obtain and use patients/consumers and family feedback | Purposefully reexamine the implementation | Stage implementation scale-up
2	Provide interactive assistance	8	31	10	7	4	4	100	Centralize technical assistance | Facilitation | Provide clinical supervision | Provide local technical assistance
3	Adapt and tailor to context	6	23	8	5	4	3	75	Promote adaptability | Tailor strategies | Use data experts
4	Develop stakeholder interrelationships	12	46	23	15	17	14	82	Build a coalition | Capture and share local knowledge | Conduct local consensus discussions | Develop academic partnerships | Identify and prepare champions | Identify early adopters | Inform local opinion leaders | Involve executive boards | Obtain formal commitments | Organize clinician implementation team meetings | Promote network weaving | Recruit, designate, and train for leadership | Use advisory boards and workgroups | Visit other sites
5	Train and educate stakeholders	23	88	63	42	11	10	91	Conduct educational meetings | Conduct educational outreach visits | Conduct ongoing training | Create a learning collaborative | Develop educational materials | Distribute educational materials | Make training dynamic | Provide ongoing consultation | Use train-the-trainer strategies | Work with educational institutions
6	Support clinicians	8	31	8	5	5	2	40	Develop resource sharing agreements | Remind clinicians
7	Engage consumers	2	8	3	2	5	2	40	Involve patients/consumers and family members | Prepare patients/consumers to be active participants
8	Change infrastructure	2	8	2	1	8	2	25	Change physical structure and equipment | Mandate change
9	Utilize financial strategies	1	4	2	1	9	2	22	Alter incentive allowance structures | Fund and contract for the clinical innovation

^a^ *ERIC* Expert Recommendations for Implementing Change

^b^ Continuous values were rounded up or down to the nearest whole number or percent.

^c^ *IS* implementation strategies

^d^ ERIC taxonomy of implementation strategies is adapted from Powell et al. [28]

Discrete and combined implementation strategies may be considered effective if they lead to improvements in *implementation outcomes*. Proctor et al. [32] defined the following eight outcomes in their Implementation Outcomes Framework (IOF) that are often perceived to be the “gold standard” outcomes in implementation research: *acceptability, adoption, appropriateness, cost, feasibility, fidelity, penetration (e.g., reach),* and *sustainability*. In other words, implementation outcomes are the effects of purposeful actions (e.g., strategies) designed to implement evidence-based or evidence-informed innovations and practices [32]. The ERIC taxonomy and IOF serve as examples of TMFs that provide a uniform language for characterizing implementation strategies and their associated implementation outcomes. These common nomenclatures help articulate implementation-related phenomena explanations, leading to an enhanced understanding of the relationship between implementation strategies and implementation outcomes [33]. As such, fields that have recently adopted implementation science principles—such as occupational therapy—should make a concentrated effort to frame their research methodologies using established implementation TMFs.

Although implementation research has seen significant progress in recent years, findings are only beginning to emerge specific to the allied health professions (e.g., OT) [24]. Implementation strategies such as educational meetings, audit and feedback techniques, and the use of clinical reminders hold promise for increasing the use of evidence by allied health professionals [24, 34]; however, there is little guidance for how these findings can be operationalized, particularly in stroke rehabilitation. This knowledge gap is particularly concerning given the Centers for Medicare & Medicaid Services (CMS)’ recent changes in payment models that provide reimbursement based on the value of services delivered. In other words, rehabilitation settings are reimbursed according to the *quality* of services implemented (as measured by improvements in patient outcomes) rather than the *quantity* of services provided. The increased attention on patient outcomes from the policy level (e.g., CMS) warrants the immediate need for OT practitioners to implement the highest quality of interventions with patients, such as stroke survivors, to improve patient outcomes and ensure that rehabilitation services are adequately reimbursed [35, 36].

As OT practitioners aim to implement high-quality, evidence-based interventions for stroke survivors, the OT profession must have a clear understanding of the strategies that have been utilized to support the use of evidence and their reported outcomes. To do this, occupational therapy and rehabilitation researchers must articulate explanations of implementation strategies and outcomes using commonly known TMFs, as well as the ERIC taxonomy and IOF. The purpose of this review is to explore the breadth of current implementation research and identify potential gaps in how occupational therapy researchers articulate their implementation strategies and report implementation outcomes for reproducibility in other research and practice contexts. Accordingly, this scoping review will address the following objectives:

1. Synthesize the types of implementation strategies—using the ERIC taxonomy—utilized in occupational therapy research to support the use of evidence-based interventions and assessments in stroke rehabilitation.

2. Synthesize the types of implementation outcomes—using the IOF—that have been measured to determine the effectiveness of implementation strategies in stroke rehabilitation.

3. Identify additional implementation theories, models, and frameworks that have guided occupational therapy research in stroke rehabilitation.

4. Describe the influence between implementation strategies and implementation outcomes.

## Methods

The scoping review methodology was guided by Arksey and O’Malley’s scoping review framework [37] and the Preferred Reporting Items for Systematic Reviews and Meta-Analyses Scoping Review (PRISMA-ScR) reporting recommendations [38]. The review team developed an initial study protocol (unregistered; available upon request) to address the review objectives and identify the breadth of literature examining implementation strategies and outcomes in stroke rehabilitation. The first author conducted preliminary searches to assess the available literature, allowing the team to revise the search strategy and search terms consistent with the iterative nature of scoping reviews. A detailed description of the search strategy can be found in the Appendix in Table 5.

### Eligibility criteria

Studies were eligible for inclusion in the review if they (a) examined the implementation of interventions or assessments, (b) had a target population of adult (18 years and older) stroke survivors, (c) included occupational therapy practitioners, and (d) took place in the rehabilitation setting. Studies published in English between Jan 2000 and May 2020 were included as the occupational therapy profession called for immediate improvements in the use of evidence to inform practice at the turn of the millennium [39], the latter date marking when the authors began the bibliographic database search. The “rehabilitation setting” was defined as acute care hospitals and post-acute care home health agencies, skilled nursing facilities, long-term acute care hospitals, hospice, inpatient rehabilitation facilities and units, and outpatient centers. Studies were excluded if they (a) only reported on intervention effectiveness (not implementation strategy effectiveness), (b) assessed psychometrics, (c) were not available in English, (d) examined pediatric patients, (e) were published as a review or conceptual article, and (f) failed to include occupational therapy practitioners as study participants.

### Information source and search strategy

The following five electronic databases were accessed to identify relevant studies in the health and mental health fields: PubMed, CINAHL, Scopus, Google Scholar, and PsychINFO. *Implementation Science* and *Implementation Science Communications* were also hand searched, as they are the premier peer-reviewed journals in dissemination and implementation research. Given the diverse terminology used to describe implementation strategies in the stroke rehabilitation field, we developed an extensive list of search terms based on previous scoping reviews that have assessed the breadth of implementation research in rehabilitation. The most recent search was conducted in May 2020. Sample search term combinations included “knowledge translation”[All Fields] OR “implement*”[All Fields]) AND “occupational therap*”[All Fields] AND “stroke”[MeSH Terms] OR “stroke” (see Additional file 1 for the complete terminology list and a database search sample). All studies identified through the search strategy were uploaded into Covidence for study selection.

### Selection process

Beginning with the study title/abstract screening phase, the first and third authors (JEM and LAJ) applied the inclusion and exclusion criteria to all studies that were identified in the initial search (agreement probability = 0.893). When authors disagreed during title/abstract screening, the second author (JLP) decided on studies to advance to the full-text review phase. Similar to scoping review screening methods conducted in the implementation science field [40], all authors reviewed a random sample (15%) of the full-text articles in the full-text screening phase to decide on study inclusion and evaluate consistency in how each author applied the inclusion/exclusion criteria. The authors achieved 100% agreement and proceeded with screening each full-text article individually.

### Data charting—extraction process

An adapted version of Arksey and O’Malley’s data charting form was created to extract variables of interest from each included study. In the data extraction phase, all authors extracted data from another random 15% of included studies to pilot test the charting form and confirm the final variables to be extracted. Authors met biweekly to share progress on independent data extraction and compare the details of data extracted across authors. Variables were extracted that represented study design, population, setting, guiding frameworks, and the description of the intervention/assessment being implemented; however, the review's primary aim was extracting information relative to implementation strategies and associated implementation outcomes.

To do this, a two-step process to extract data on implementation strategies and outcomes was used. In Step 1, team members charted the specific terminology used to describe strategies or outcomes in each study. In Step 2, the review team used a directed content analysis approach to map this charted information and terminology to the ERIC taxonomy [28] and the IOF [32]. For instance, an implementation strategy that authors initially described as “holding in-services with clinicians” was “translated” to “conducting educational meetings.” Likewise, implementation outcomes that were initially described as “adherence” were converted to “fidelity.” This translation process was guided by descriptions of implementation strategies as listed in the original 2015 ERIC project publication (as well as the ERIC ancillary material) and the seminal IOF publication from 2011. The extracted and translated data was entered using the Excel for Microsoft 365 program.

### Synthesis process

The authors followed Levac et al.’s [41] recommendations for advancing scoping methodology to synthesize data. One author (JEM) cleaned the data (e.g., spell check, cell formatting) to ensure that Excel accurately and adequately performed operations, calculations, and analyses (e.g., creating pivot tables, charts). As scoping reviews do not seek to aggregate findings from different studies or weigh evidence [37, 41], only descriptive analyses (e.g., frequencies, percentages) were conducted from the extracted data to report the characteristics of the included studies and thematic clusters. The descriptive data and results of the directed content analysis were organized into tables using themes to articulate the review’s findings that addressed the research objectives.

## Results

**Fig. 1:**
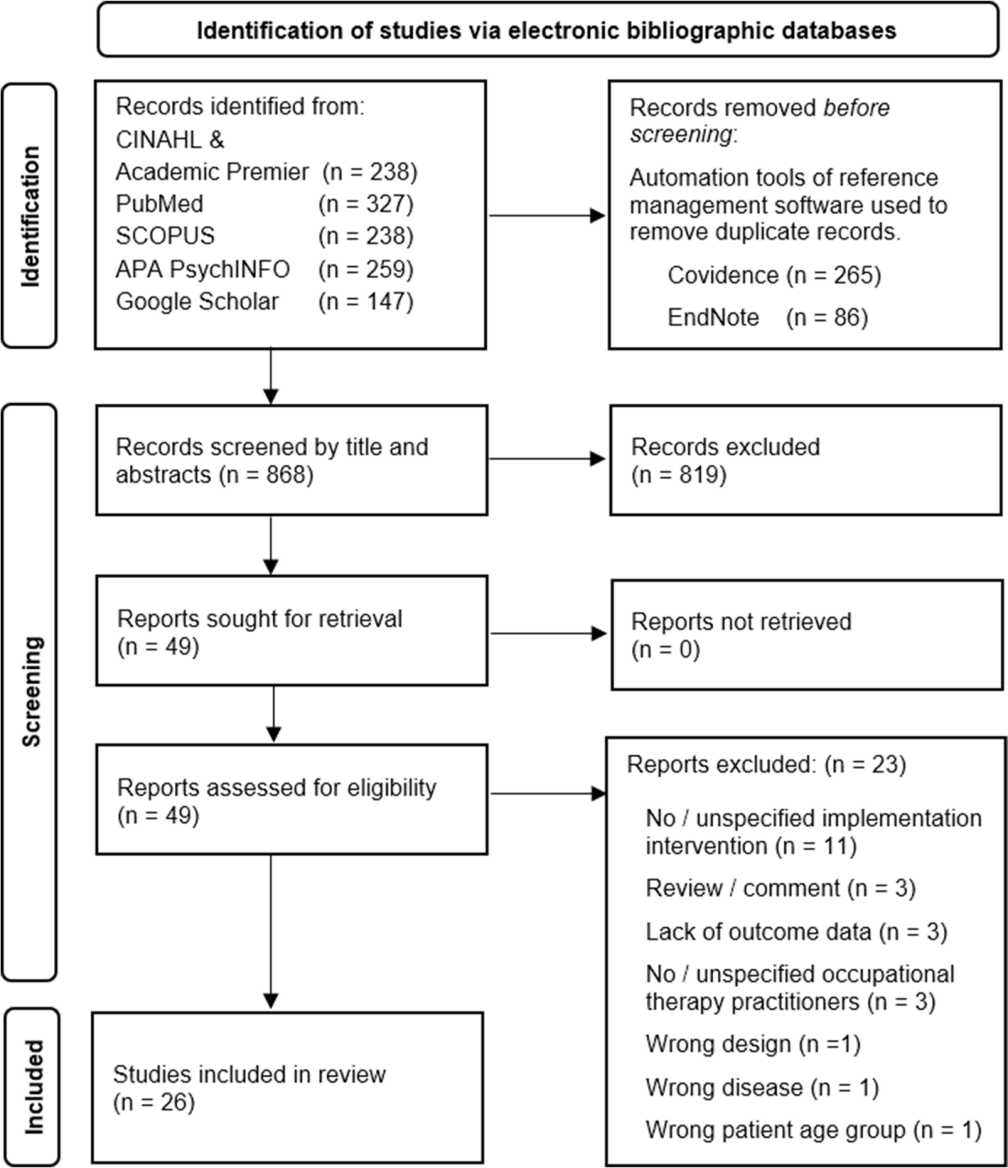
PRISMA flow diagram [42] outlining the review’s selection process

The search yielded 1219 articles. After excluding duplicates, 868 titles and abstracts were reviewed for inclusion. Among those, 49 articles progressed to full-text review, and 26 met the criteria for data extraction, as shown in Fig. 1.

### Study characteristics

Table 2 describes the studies’ characteristics. The studies were published between 2005 and 2020, all within the last 10 years except one [43]. Studies were most set in Australia (27%) and most commonly conducted in an inpatient rehabilitation healthcare setting (65%). While two studies targeted practitioners in any healthcare setting by implementing an educational related implementation strategy (e.g., conduct ongoing training) either at an offsite location [44] or nonphysical [45] environment, none of the studies was conducted in a long-term acute care hospital (LTACH) or hospice setting. Most studies used a pre–post research design (50%), followed by process evaluation (14%). Studies used quantitative methods (69%) most frequently, with similar utilization between qualitative (12%) and mixed-method (19%) approaches. While the studies primarily implemented stroke-related interventions (92%), this was not mutually exclusive, as some implemented a combination of an intervention (e.g., TagTrainer), an assessment (e.g., Canadian Occupational Performance Measure (COPM)), or clinical knowledge (e.g., upper limb poststroke impairments).

**Table 2:** Study characteristics (*N* =26)

Country	Sample size (***n***)	Percentage	Method	Sample size (***n***)	Percentage	Study design	Sample size (***n***)	Percentage	Healthcare setting^a^	Sample size (***n***)	Percentage	Innovation^a^	Sample size (***n***)	Percentage
Australia	7	27	Quantitative	18	69	Cross-sectional	1	4	Acute care	7	27	Assessment	4	15
Canada	8	31	Qualitative	3	12	Formative evaluation	1	4	Community	7	27	Intervention	24	92
Denmark	1	4	Mixed methods	5	19	Hermeneutic phenomenology	1	4	Home health	2	8	Knowledge	10	38
Netherlands	3	12				Longitudinal	1	4	Inpatient rehabilitation^b^	17	65			
Sweden	1	4				Participatory action research	1	4	Long-term care	1	4			
Uganda	1	4				Pre–post	13	50	Outpatient^c^	6	23			
The UK	2	8				Process evaluation	4	15	Skilled nursing	1	4			
The USA	3	12				Prospective cohort	1	4	Other^d^	3	12			
						Randomized controlled trial	1	4						
						Retrospective cohort	1	4						
						Time series	1	4						

*N* = 26 unless specified otherwise

Percentages were rounded up or down to the nearest whole number.

^a^ Responses are not mutually exclusive

^b^ Inpatient rehabilitation includes freestanding inpatient rehabilitation hospitals (or facilities) and units and was identified in studies as IRF, acute rehabilitation, rehabilitation hospital, or rehabilitation center

^c^ Outpatient includes all outpatient clinics regardless of specialty (e.g., outpatient hand therapy clinic)

^d^ Other includes studies that listed “other” as a healthcare setting or a study involved disseminating knowledge to practitioners working in any healthcare setting, and education was provided at an offsite location or nonphysical environment (e.g., workshop, or online training)

### Implementation strategies

The studies included in this review collectively utilized 48 of the 73 discrete strategies drawn from the ERIC taxonomy. Discrete implementation strategies per study ranged from 1 to 21, with a median of four strategies used per study. The two most commonly used implementation strategies applied in 42% of studies were *distribute educational materials* [44, 46–55] and *assess for readiness and identify barriers and facilitators* [47–49, 52, 56–62]*.* The latter strategy implies two separate actions; however, only two studies [48, 49] *assessed readiness “*and” *identified barriers and facilitators.* Other discrete implementation strategies frequently used included: *conduct educational outreach visits, conduct ongoing training, audit & provide feedback*, and *develop educational materials.* Of all studies included in this review, 88% used at least one of these six primary strategies.

#### Thematic clusters of implementation strategies

Waltz et al. [29] identified nine thematic clusters using the ERIC taxonomy (Table 1), which allowed further exploration of another dimension of the implementation strategies. Table 1 provides a summary of how the implementation strategies were organized in terms of thematic clusters. Twenty three of the 26 studies [43, 44, 46–60, 62–67] implemented at least one discrete implementation strategy in the cluster, *train and educate stakeholders*, followed by 17 of 26 studies which examined strategies in the *use evaluative and iterative strategies* cluster [47–50, 52, 55–62, 65–68]. The *train and educate stakeholders* cluster comprises four of the six most used implementation strategies: *conduct ongoing training, develop educational materials, conduct educational outreach visits,* and *distribute educational materia*l*.* The other two commonly used implementation strategies, *assess for readiness and identify barriers and facilitators* and *audit and provide feedback,* are categorized in the cluster *use evaluative and iterative strategies.*

Within the *change infrastructure* cluster*,* one study used the implementation strategy *mandate change* [50], and another study used *change physical structure & equipment* [65]. Within the cluster of *utilize financial strategies*, one study [50] used the following implementation strategies: *alter incentive/allowance structure* and *fund & contract for the clinical innovation*. The included studies applied the least number of strategies from this cluster, with only two out of the nine possible implementation strategies being used—the lowest percentage, 1%, used amongst the thematic clusters.

### Implementation outcomes

Table 3 provides a summary of the measurements and implementation outcomes used in each study. The implementation outcomes measured per study ranged from 1 to 4. Studies most frequently included two implementation outcomes, with *adoption* being frequently measured in 81% of studies [43–45, 48–55, 57, 59–64, 66–68]. *Fidelity* followed and was measured in 42% of studies [43, 47, 52, 53, 56–58, 60, 63, 65, 68]. Seven of the eight implementation outcomes were measured in at least one of the studies, whereas *implementation cost* was the only implementation outcome not addressed in any of the studies. Moreover, Moore et al.’s [50] study is the only one to measure *penetration* and *sustainability*. All the studies used various approaches to measuring implementation outcomes, as shown in Table 3. For example, 11 of 20 studies measuring *adoption* used administrative data, observations, or qualitative or semi-structured interviews [43, 52, 53, 55, 57, 59, 60, 62–64, 66, 68].

**Table 3:** Summary of data for studies included in the review

Author(s)	Study design	Implementation strategy	Implementation outcome	Outcome measurement	Related findings
Bland et al. [56]	Retrospective cohort	1) Assess for readiness and identify barriers and facilitators 2) Audit and provide feedback 3) Conduct educational meetings 4) Develop and implement tools for quality monitoring	Fidelity	Visual inspection of 17 months of time series for increased adherence > 5%	Median adherence ranged from .52 to .88 across all settings and professional disciplines; PT had the greatest adherence across disciplines (*p* < .004); IRF and acute higher had adherence than outpatient (*p* < .001). Specific events increased adherence 40% of the time, with those gains maintained for >1 month 60% of the time.
Braun et al. [46]	Process evaluation	1) Conduct educational outreach visits 2) Distribute educational materials 3) Identify and prepare champions 4) Make training dynamic	Appropriateness Feasibility	Pre structured patient files; patient logs; therapist and patient questionnaires	In 11 out of 16, 69%, of participants, the mental practice intervention was delivered according to the framework; patients received the minimum amount of mental practice recommended (13 out of 14, 93%), and they undertook unguided (12 out of 16, 75%) practice as recommended. Implementation was more challenging than expected.
Clarke et al. [62]	Process evaluation	1) Assess for readiness and identify barriers and facilitators 2) Conduct educational meetings 3) Use train-the-trainer strategies	Adoption	Ethnographic approach—observations, interviews & documentary analysis	Minimal adoption of LSCTC across units and professions; adoption varied due to staff skill and expertise, infrastructure, and local mgmt. factors. Contextual factors, including organizational history and team relationships, external policy, and service development initiatives, impinged on the implementation of the caregiver training program in unintended ways.
Connell et al.^1^ [63]	Formative evaluation	1) Capture and share local knowledge 2) Create a learning collaborative 3) Work with educational institutions	Adoption Fidelity	Semi-structure interviews	Fidelity to GRASP was lower than expected (not always used in a way shown to be effective) even though adoption was perceived to be strong. Almost all intervention components were adapted to some degree when used in clinical practice; coverage was wider, the content adapted, and the dose, when monitored, was less.
Connell et al.^2^ [45]	Cross-sectional	1) Identify early adopters 2) Visit other sites	Acceptability Adoption Appropriateness Feasibility	Self-administrated questionnaire	Sixty-one therapists (22.2%) reported that they had tried occasionally or regularly in practice (33 PTs & 28 OTs). GRASP was used most frequently by therapists in community settings (*n* = 27), followed by rehabilitation (*n* = 20) and acute (*n* = 14). Therapists working in rehabilitation were significantly more positive when asked about whether a manual would have positive outcomes for people with stroke (*p* = .003), the applicability of a manual in stroke rehabilitation (*p* = .009), and whether it could be easily incorporated into their work setting (*p* = .002). Therapists displayed positive opinions toward implementing a manual with graded progressions of structured upper limb exercises for people after stroke. Opinions were different between therapists who had used GRASP and those who had not.
Doyle and Bennett [44]	Pre–post	1) Conduct educational outreach visits 2) Develop educational materials 3) Distribute educational materials	Acceptability Adoption	Questionnaire; Patient Practitioner Orientation Scale (PPOS)	A theory-based workshop yielded significant changes in knowledge, attitudes, and perceived behavioral control and intended behaviors about sensory impairment management, research utilization, and shared decision making. Preworkshop: agreement for current behaviors ranged 5.3–52.6%, knowledge scores (*M* = 3.32, *SD* = 1.06), PPOS scores (*M* = 54.56, *SD* = 6.39). Postworkshop: agreement for intended behaviors ranged 84.2 to 100%, knowledge scores (*M* = 8.53, *SD* = 0.91) statistically significant (*p* = .00), PPOS scores (*M* = 49.22, *SD* = 5.80) statistically significant (*p* = .00)—indicating more patient-centeredness.
Eriksson et al. [55]	Longitudinal	1) Create a learning collaborative 2) Develop academic partnerships 3) Develop educational materials 4) Distribute educational materials 5) Facilitation 6) Make training dynamic 7) Obtain formal commitments 8) Organize clinician implementation team meetings 9) Promote adaptability 10) Purposefully reexamine the implementation	Adoption	Focus group interviews	Three subcategories were identified from the focus group interviews: (1) including in the scientific world, (2) involving as an actor of science, and (3) integrating into a partnership. A core category emerged: the implementation of client-centered practice enabled the fusion of practice and science. An increased experience of using CADL and support from the researchers changed the OTs' attitudes towards engaging in research from being an outsider to the scientific world to being included and then becoming a part of the research as an implementer of science.
Frith et al. [54]	Pre–post	1) Develop educational materials 2) Develop resource sharing agreements 3) Distribute educational materials 4) Tailor strategies	Adoption Acceptability	Knowledge test and survey	Two hundred four learners completed the module: 68% of learners scoring 100% in the post-module knowledge test. It was not possible to determine whether a behavior change had occurred as a result of completing the RTD module or whether this had translated to improved care in the management of RTD. Twelve learners completed the additional survey (8 OTs)—self-report outcomes indicate positive effects taking more responsibility (*n* = 8) in managing RTD, providing clearer information to patients (*n* = 4), and supplementing verbal information with written information.
Kristensen and Hounsgaard [68]	Hermeneutic phenomenology	1) Audit and provide feedback	Adoption Fidelity	OT medical record; daily self-reported recordings; focus group interviews	Audit and feedback methods proved useful for providing therapists with important information to evaluate and further the implementation process. Daily practice in both settings adapted to the clinical guidelines. Implementations of the standardized assessment tools (AMPS, A-ONE, COPM) seemed to be the most successful.
Levac et al.^1^ [64]	Pre–post	1) Develop educational materials 2) Identify and prepare champions 3) Make training dynamic 4) Provide ongoing consultation 5) Remind clinicians	Adoption Appropriateness	Focus group interviews; ADOPT-VR instrument; self-reported knowledge and skills survey	The therapist's intention to use VR did not change. Knowledge and skills improved significantly following e-learning completion (*p* = 0.001) and were sustained six months post-study. Below average perceived usability of the IREX (19th percentile) was reported. Lack of time was the most frequently reported barrier to VR use. A decrease in the frequency of perceived barriers to VR use was not significant (*p* = 0.159). Therapists reported that client motivation to engage with VR facilitated IREX use in practice but that environmental and IREX-specific barriers limited use.
Levac et al.^2^ [53]	Pre–post	1) Conduct educational outreach visits 2) Distribute educational materials 3) Remind clinicians	Adoption Fidelity	Self-report survey; focus group interviews	The KT intervention improved self-reported confidence about MLS use as measured by confidence ratings (*p* < 0.001). Therapists favored transferring skills from VR to real-life tasks over employing a more comprehensive MLS approach. Chart-Stimulated Recall indicated a moderate level of competency in therapists' clinical reasoning about MLSs following the intervention, with no changes following additional opportunities to use VR (*p* = .944). No behavior change for MLS use was noted (*p* = 0.092) on the Motor Learning Strategy Rating Instrument
Luconi et al. [61]	Prospective cohort	1) Assess for readiness and identify barriers and facilitators 2) Identify and prepare champions 3) Remind clinicians	Appropriateness Feasibility	My Guidelines Implementation Barometer (MGIB); questionnaire; comments; Information Assessment Method (IAM)	Satisfaction, relevance, and cognitive impact of delivered information varied across disciplines and recommendations. Agreement with the recommendations was high across disciplines. On average, three interdisciplinary recommendations (related to post-stroke depression, post-stroke fatigue, and patients' and caregivers’ learning needs) were rated the most relevant for at least one patient. Most clinicians would use the recommendations for a specific patient and expected health benefits by applying those recommendations.
McCluskey and Middleton [59]	Pre–post	1) Assess for readiness and identify barriers and facilitators 2) Audit and provide feedback 3) Conduct educational outreach visits	Adoption Feasibility	Administrative data (medical records)	Medical record audits found that teams delivered six or more outdoor journeys to 17% of people with stroke pre-intervention, rising to 32% by 12 months post-intervention. This change represents a modest increase in practice behavior (15%) across teams. The “Out-and-About Implementation Program” helped rehabilitation teams change their practice, implement evidence, and improve client outcomes.
McCluskey et al.^1^ [60]	Pre–post	1) Assess for readiness and identify barriers and facilitators 2) Build a coalition 3) Conduct educational outreach visits 4) Develop educational materials 5) Identify and prepare champions 6) Provide clinical supervision	Adoption Feasibility Fidelity	Administrative data; motor assessment scale; box and block test; nine hole peg test; motor activity log	Sixteen stroke participants were recruited (*M* = 15.3 months post-stroke, *SD* 11.9), and 6 CIMT programs were conducted over 24 months, compared with none pre-implementation. The behavior change program resulted in multiple CIMT programs being delivered safely and with fidelity. Capacity building, skill development, and preparation for the first CIMT program took many hours.
McCluskey et al.^2^ [52]	Randomized controlled trial	1) Assess for readiness and identify barriers and facilitators 2) Audit and provide feedback 3) Conduct ongoing training Distribute educational materials	Adoption Fidelity	Administrative data	At 12 months after implementing the behavior change program, 9% of audited experimental group stroke survivors received four or more outings during therapy compared with 5% in the control group (adjusted risk difference 4%, 95% CI [9 to 17], *p* = 0.54). The Out-and-About program did not change team or stroke survivor behavior.
McEwen et al.^1^ [43]	Pre–post	1) Conduct educational meetings 2) Conduct educational outreach visits 3) Develop educational materials 4) Facilitation 5) Provide technical assistance 6) Provide ongoing consultation 7) Recruit, designate, and train for leadership	Adoption Fidelity	Questionnaire; administrative data (medical records)	No charts showed evidence of CO-OP use at baseline, compared with 8/40 (20%) post-intervention. Post-intervention, there was a trend towards reduction in impairment goals, and significantly more component goals were set (*z* = 2.7, *p* = .007)
McEwen et al.^2^ [51]	Pre–post	1) Centralize technical assistance 2) Conduct educational outreach visits 3) Conduct ongoing training 4) Distribute educational materials 5) Identify and prepare champions 6) Promote network weaving 7) Remind clinicians	Adoption	Written tests; surveys	Participation in REPS was associated with an increase in stroke rehabilitation knowledge immediately following the program and at 6-month follow-up; participants reported positive practice changes following completion of the program and at the 6-month follow-up
Moore et al. [50]	Time series	1) Alter incentive allowance structures 2) Audit and provide feedback 3) Conduct local consensus discussions 4) Conduct ongoing training 5) Develop academic partnerships 6) Develop and organize quality monitoring systems 7) Develop educational materials 8) Distribute educational materials 9) Fund and contract for the clinical innovation 10) Identify and prepare champions 11) Involve executive boards 12) Mandate change 13) Promote adaptability 14) Provide clinical supervision 15) Provide ongoing consultation 16) Purposefully reexamine the implementation 17) Stage implementation scale-up 18) Tailor strategies 19) Use advisory boards and workgroups 20) Use data experts 21) Use train-the-trainer strategies	Adoption Penetration Sustainability	Surveys	Survey data indicate the BRAI resulted in a significant increase in the use of EBPs to make clinical decisions and justify care. Survey participants reported a substantial increase in the use of outcome measures in 2012 (74%) and 2015 (91%) and EBP in 2012 (62%) and 2015 (82%). In 2012, significant differences (*p* = .01) in the effect of the BRAI on practice were identified between therapists who were directly involved in the project and interventions compared with uninvolved therapists. In 2015, no significant differences existed between involved and uninvolved therapists. After 6 years of sustained implementation efforts, the BRAI expedited the adoption of EBPs throughout a large system of care in rehabilitation.
Petzold et al [49]	Pre–post	1) Assess for readiness and identify barriers and facilitators 2) Conduct educational outreach visits 3) Conduct ongoing training 4) Develop educational materials 5) Distribute educational materials 6) Make training dynamic 7) Remind clinicians 8) Tailor strategies	Adoption Feasibility Acceptability	Knowledge questionnaire; EBP self-efficacy scale; clinician/work environment variables measure; patient case vignettes	A significant improvement in knowledge of best practice unilateral spatial neglect management (*p* < 0.000) and evidence-based practice self-efficacy in carrying out evidence-based practice activities (*p* < 0.045) post-intervention.
Salbach et al. [48]	Process evaluation	1) Assess for readiness and identify barriers and facilitators 2) Capture and share local knowledge 3) Conduct educational outreach visits 4) Create a learning collaborative 5) Distribute educational materials 6) Facilitation 7) Identify and prepare champions 8) Remind clinicians	Adoption	Patient outcomes; self-report checklists	Facilitated KT intervention was associated with improved implementation of sit-to-stand (*p* = 0.028) and walking (*p* = 0.043) training. In contrast, the passive KT intervention was associated with improved implementation of standing balance training (*p* = 0.037) after adjusting for clustering at patient and provider levels and covariates. Facilitated KT intervention was unsuccessful in improving the integration of 18 treatments concurrently. The facilitated approach may not have adequately addressed barriers to integrating numerous treatments simultaneously and complex treatments that were unfamiliar to providers
Schneider et al. [58]	Pre–post	1) Assess for readiness and identify barriers and facilitators 2) Conduct educational meetings 3) Create a learning collaborative 4) Develop a formal implementation blueprint	Fidelity	Observations; recorded data	Outcomes were measured across *n* = 15 classes (*n* = 30 patients). Between baseline and 12 months, the mean proportion of practice time per class increased by 52% (95% *CI* 33–70; *p* < 0.001), and the mean no. of repetitions per practice time increased by 5.1 reps/min (95% *CI* 1.7–8.4; *p* < 0.01). Between baseline and 18 months, the mean proportion of practice time per class increased by 53% (95% *CI* 36–69; *p* < 0.001), and the mean no. of reps per practice time increased by 3.9 reps/min (95% *CI* 1.9–5.9; *p* < 0.001). Providing professional development was associated with increased intensity of practice in an inpatient, upper limb rehabilitation class. The increase was maintained 6 months later.
Stewart et al. [57]	Pre–post	1) Assess for readiness and identify barriers and facilitators 2) Audit and provide feedback 3) Conduct ongoing training 4) Create a learning collaborative 5) Provide ongoing consultation	Adoption Fidelity	Medical record audit; behavioral mapping; observations	Post-intervention, no. of participants with practice books increased from 1 to 6 (*OR* = 11, 95% *CI* = (0.9, 550.7)), but this change was not statistically significant (*p* = 0.069). There was no change in median repetitions recorded (*r* = 0.00, 95% *CI* = (− 0.4, 0.4), *p* = 1.000) or observed active practice (*r* = − 0.02, 95% *CI* = (− 0.4, 0.4), *p* = 0.933). The staff behavior change intervention led to increasing use of practice books but no statistically significant difference in adoption of practice books or intensity of active practice.
Terio et al. [65]	Process evaluation	1) Audit and provide feedback 2) Change physical structure and equipment 3) Conduct educational outreach visits 4) Conduct ongoing training 5) Develop a formal implementation blueprint 6) Facilitation 7) Involve patients/consumers and family members 8) Promote adaptability 9) Provide local technical assistance 10) Purposefully reexamine the implementation	Acceptability Fidelity	Logbooks; semi-structure interviews	In 11 out of 14 cases, the clients were compliant with the intervention. However, challenges such as technical problems were reported. The target of conducting 16 phone calls for each client was achieved to 74%. Mechanisms contributing to the implementation of the intervention included engaged facilitators and motivated participants. Challenges in client recruitment and poor information dissemination were some of the mechanisms impeding the implementation. Several mediators in the process drove the project forward, including strong facilitation and motivated participants.
Tetteroo et al. [66]	Participatory action research	1) Conduct educational meetings 2) Conduct ongoing training 3) Make training dynamic 4) Provide local technical assistance 5) Provide ongoing consultation 6) Purposefully reexamine the implementation	Adoption Acceptability	Semi-structure interviews; questionnaires; observation notes; usage logs	TagTrainer system was used in 34 therapy sessions, 20-group, 14-individual. In general, therapists reported moderate to high self-efficacy, except for their perceived ability to resolve technical problems with TagTrainer (*M* = 32.5, *SD* = 28.7). In addition, they reported significantly higher levels of self-efficacy (*t*(3)=4.899, *p* = 0.016) for using TagTrainer in individual therapy sessions (*M* = 80.0, *SD* = 21.6), compared with group therapy sessions (*M* = 60.0, *SD* = 28.3). The credibility (*M* = 19.5, *SD* = 3.11) and expectancy (*M* = 13.9, *SD* = 5.22) ratings that the therapists gave for the TagTrainer system show that they find it to be credible for arm–hand rehabilitation but are neutral in respect to the expected effectiveness of the system for the improvement of arm–hand performance.
Vratsistas-Curto et al. [47]	Pre–post	1) Assess for readiness and identify barriers and facilitators 2) Audit and provide feedback 3) Conduct cyclical small tests of change 4) Conduct educational meetings 5) Conduct ongoing training 6) Distribute educational materials 7) Provide ongoing consultation	Fidelity	Medical records; administrative data	Between the 1st & 4th audits (2009 & 2013), 20 of the 27 areas targeted (74%) met or exceeded the minimum target of 10% change. Practice areas that showed the most change included sensation screening (+ 75%) and rehabilitation (+ 100%), neglect screening (+ 92%), and assessment (100%). Some target behaviors showed a drop in compliance, such as anxiety and depression screening (− 27%) or little or no overall improvement, such as patient education about stroke (6% change). Audit feedback and education increased the proportion of inpatients with stroke receiving best practice rehabilitation in some but not all practice areas.
Willems et al. [67]	Pre–post	1) Inform local opinion leaders 2) Involve patients/consumers and family members 3) Obtain and use patients/ consumers and family feedback 4) Prepare patients/consumers to be active participants 5) Promote adaptability 6) Recruit, designate, and train for leadership 7) Remind clinicians 8) Stage implementation scale-up 9) Use train-the-trainer strategies	Adoption	Questionnaires	After the knowledge broker (KB) intervention, more patients (48%; *n* = 217) reported at least some encouragement by HPs to be physically active than before (26%; *n* = 243, *p* < 0.000). HPs (*n* = 288) on average reported encouraging patients more often after the intervention, but this difference was significant only for occupational therapists and KBs. Based on the patient’s reports of HP behavior, the KB intervention appears effective since more patients felt encouraged to be physically active after the intervention than before.

The naming convention for discrete implementation strategies is adapted from the ERIC taxonomy of implementation strategies [28], and the naming convention for implementation outcomes is adapted from Proctor et al.’s Taxonomy of Implementation Outcomes [32]

^1, 2^ Identifies a reference citation for two seperate articles that share similar or the same authors

### Theories, models, and frameworks

Notably, of the 26 included articles, 12 explicitly stated using a TMF to guide the selection and application of implementation strategies (Table 4). The most common supporting TMF employed among the articles (*n* = 5) was the Knowledge-to-Action Process framework [44, 48–50, 61], categorized as a process model. Classic or classic change theory was the next most commonly applied category of TMFs, including the Behavior Change Wheel [47, 57, 60] (*n* = 3) and Theory of Planned Behavior [44] (*n* = 1). No implementation evaluation frameworks were utilized (e.g., Reach, Efficacy, Adoption, Implementation, Maintenance (RE-AIM) or Implementation Outcomes Framework). A select number of studies described the components of their implementation strategies following reporting guidelines. Two studies [47, 64] used the Template for Intervention Description and Replication (TIDieR) checklist. One study [47] used the Standards for Quality Improvement Reporting Excellence (SQUIRE). Moreover, one study [57] followed the Standards for Reporting Implementation studies (StaRI) checklist but did not explicitly mention an implementation framework to guide study design.

**Table 4:** Summary of implementation theories, models, and frameworks (TMF**s**) used in studies

Author(s)	Year	Usage (Y/N)	Implementation TMF^a^	Category of TMF^a, b^
McEwen et al.^1^ [43]	2005	No		
Braun et al. [46]	2010	No		
McCluskey and Middleton [59]	2010	No		
Petzold et al. [49]	2012	Yes	Knowledge-to-Action (KTA) Process Framework	Process model
Bland et al. [56]	2013	No		
Clarke et al. [62]	2013	Yes	Normalization Process Theory	Implementation theory
Connell et al.^1^ [63]	2014	Yes	Normalization Process Theory	Implementation theory
Connell et al.^2^ [45]	2014	Yes	Consolidated Framework for Implementation Research (CFIR)	Determinant framework
Doyle and Bennett [44]	2014	Yes	Knowledge-to-Action (KTA) Process Framework	Process model
			Theory of Planned Behavior	Classic (or classic change) theory
Kristensen and Hounsgaard [68]	2014	No		
Tetteroo et al. [66]	2014	No		
Levac et al.^1^ [64]	2016	No		
Levac et al.^2^ [53]	2016	No		
McCluskey et al.^2^ [52]	2016	No		
Willems et al. [67]	2016	No		
Eriksson et al. [55]	2017	No		
Frith et al. [54]	2017	No		
Salbach et al. [48]	2017	Yes	Knowledge-to-Action (KTA) Process Framework	Process model
Vratsistas-Curto et al. [47]	2017	Yes	Theoretical Domains Framework	Determinant framework
			Behavior Change Wheel	Classic (or classic change) theory
Moore et al. [50]	2018	Yes	Knowledge-to-Action (KTA) Process Framework	Process model
McEwen et al.^2^ [51]	2019	No		
Schneider et al. [58]	2019	No		
Terio et al. [65]	2019	Yes	Integrated Promoting Action on Research Implementation in Health Services (i-PARIHS)	Determinant framework
Luconi et al. [61]	2020	Yes	Knowledge-to-Action (KTA) Process Framework	Process model
McCluskey et al.^1^ [60]	2020	Yes	Behavior Change Wheel	Classic (or classic change) theory
Stewart et al. [57]	2020	Yes	Behavior Change Wheel	Classic (or classic change) theory

^a^ *TMFs* theories, models, and frameworks

^b^ Taxonomy of categories of theories, models, and frameworks adapted from Nilsen [69]

^1,2^ Identifies a reference citation for two seperate articles that share similar or the same author

### Association between implementation strategies and implementation outcomes

The findings from studies examining the effect of implementation strategies on implementation outcomes were generally mixed. While 42% of studies used strategies that led to improved implementation outcomes, 50% led to inconclusive results. For instance, McEwen et al. [51] developed a multifaceted implementation strategy that involved conducting educational meetings, providing ongoing education, appointing evidence champions, distributing educational materials, and reminding clinicians to implement evidence in practice. These strategies led to increased adoption of their target EBP, the Cognitive Orientation to daily Occupational Performance (CO-OP) treatment approach, suggesting this multifaceted strategy may facilitate EBP implementation among OTs. Alternatively, Salbach et al. [48] examined the impact of an implementation strategy consisting of educational meetings, evidence champions, educational materials, local funding, and implementation barrier identification that pertained to stroke guideline adoption. However, these strategies only led to the increased adoption of two out of 18 recommendations described in the stroke guidelines. Levac et al. [64] also utilized a combination of educational meetings, dynamic training, reminders, and expert consultation to increase the use of virtual reality therapy with stroke survivors, yet found these combined strategies did not lead to an increase in virtual reality adoption among practitioners serving stroke survivors.

## Discussion

This scoping review is the first to examine implementation strategy use, implementation outcome measurement, and the application of theories, models, and frameworks in stroke rehabilitation and occupational therapy. Given that implementation science is still nascent in occupational therapy, this review’s purpose was to synthesize implementation strategies and outcomes using uniform language—as presented by the ERIC and IOF taxonomies—to clearly understand the types of strategies being used and outcomes measured in the occupational therapy and stroke rehabilitation fields. Importantly, this review also calls attention to the value of applying theories, models, and frameworks to guide implementation strategy selection and implementation outcome measurement.

Operationalizing implementation strategies and outcomes are essential for reproducibility in subsequent research studies and in practice. Without a clear language for defining strategies and reported outcomes, stroke rehabilitation and occupational therapy researchers place themselves at risk of contributing to what is currently being referred to as the “secondary” research-to-practice gap. This secondary gap is emerging in implementation science because empirical findings from implementation science have seldom been integrated into clinical practice [70]. For instance, the present review found that the distribution of educational materials was one of the most commonly utilized implementation strategies, yet it has been well established that educational materials alone are typically insufficient for changing clinical practice behaviors [71]. One potential reason that may explain why implementation science discoveries are rarely integrated into real-world practice may pertain to the fact that implementation strategies and outcomes are not consistently named or described, leading to difficulties replicating these strategies in real-world contexts. Using the ERIC and IOF to guide the description of strategies and reported outcomes is a logical first step in enhancing the replication of effective strategies for improving implementation outcomes.

Further, replication can be enhanced by describing strategies according to specification guidelines. Four studies in this review described implementation strategies using reporting standards such as the Template for Intervention Description and Replication (TIDieR) checklist, the Standards for Quality Improvement Reporting Excellence (SQUIRE), and Standards for Reporting Implementation studies (StaRI). Though the use of these reporting standards is promising for optimizing replication, Proctor et al. [27] also provide recommendations for how to specify implementation strategies designed to improve specific implementation outcomes. These recommendations include clearly *naming* the implementation strategy, *describing* it, and *specifying* the strategy according to the following parameters: *actor*, *action*, *action target*, *temporality*, *dose*, *outcome affected*, and *justification*. These recommendations have been applied in the health and human services body of literature [72, 73], but their application remains scarce in the fields of rehabilitation and occupational therapy [74].

One noteworthy finding from this review was the variation with which studies were guided by implementation TMFs. Fewer than half of the studies (*n* = 12) were informed by TMFs drawn from the implementation literature. The Knowledge-to-Action Process framework was applied in five studies, followed by the Behavior Change Wheel and Normalization Process Theory, represented in three and two studies, respectively. The lack of TMF application may also explain some of the variability in implementation strategy effectiveness. Interestingly, all 12 studies with TMF underpinnings found either mixed or beneficial outcomes as a result of their implementation strategies.

Conversely, the three studies that found no effect of their strategies on implementation outcomes were not informed by any implementation TMF. While this subset of studies is too small to draw definitive conclusions, the importance of using TMFs to guide implementation studies have been well established and endorsed by leading implementation scientists to identify the determinants that may influence implementation, understand relationships between constructs, and inform implementation project evaluations [25, 69, 75]. Despite their recognized importance, TMFs are often applied haphazardly in implementation projects, and the selection of appropriate TMFs is complicated given the proliferation of TMFs in the implementation literature [33]. While tools (e.g., dissemination-implementation.org/content/select.aspx) are available to help researchers in TMF selection, occupational therapy researchers in stroke rehabilitation who are new to the field of implementation science may be unfamiliar with such tools and resources. For instance, Birken et al. have developed the Theory, Model, and Framework Comparison and Selection Tool (T-CaST) that assesses the “fit” of different TMFs with implementation projects based on four areas: usability, testability, applicability, and acceptability [25]. Similarly, TMF experts have also developed a list of 10 recommendations for selecting and applying TMFs, and published specific case examples of how one TMF, the Exploration, Preparation, Implementation, Sustainment framework, has guided several implementation studies and projects [76].

In addition to synthesizing implementation strategies and outcomes that have been examined in the stroke rehabilitation literature, this review also corroborates other reviews in the rehabilitation field, which have found the mixed effectiveness of implementation strategies. A Cochrane review by Cahill et al. [77] was unable to determine the effect of implementation interventions on healthcare provider adherence to evidence-based practice in stroke rehabilitation due to limited evidence and lower-quality study designs. However, one encouraging finding from the present review, and specific to the occupational therapy field, was the frequent use of the following implementation strategy: *assess for readiness and identify barriers and facilitators*. The assessment of barriers and facilitators is a central precursor to selecting implementation strategies that effectively facilitate the use of evidence in practice [78]. Implementation strategies that are not responsive to these barriers and facilitators frequently fail to produce sufficient and sustainable practice improvements [78, 79].

Although identifying implementation barriers and facilitators is of paramount importance in implementation studies, the processes researchers use to select relevant implementation strategies based on these barriers and facilitators are often unclear. Vratsistas-Curto et al. [47], for instance, assessed determinants of implementation at the start of their study and mapped determinants to the Theoretical Domains Framework and Behavior Change Wheel to inform implementation strategy selection. This exemplar use of TMFs can strengthen the rigor of implementation strategy selection and elevate strategy effectiveness. However, not all implementation studies are informed by underlying TMFs, calling into question the rationale behind why specific strategies are used in certain contexts. Going forward, as the fields of stroke rehabilitation and occupational therapy grow their interest in implementation, researchers must be transparent when explaining the process and justification of their implementation strategy selection. Without this transparency, occupational therapy stakeholders and other rehabilitation professionals may continue to use implementation strategies without systematically matching them to identified barriers and facilitators. To facilitate strategy selection, Waltz et al. [78] gathered expert opinion data and developed a tool matching implementation barriers to implementation strategies. The tool draws language from the Consolidated Framework for Implementation Research (CFIR) [23] and matches identified CFIR barriers to the ERIC taxonomy of implementation strategies. Using the CFIR-ERIC matching tool may be a viable option for occupational therapy and stroke rehabilitation researchers who understand determinants of evidence implementation but require guidance when selecting relevant implementation strategies.

The other commonly examined implementation strategy identified in this review involved the use of educational meetings and materials. Eleven studies used one or more of these educational techniques to facilitate the implementation of evidence into practice. However, in the context of these educational techniques, all studies examining educational strategies failed to specify their implementation strategies as recommended by reporting guidelines [27]. Perhaps this lack of strategy specification can be attributed to the interdisciplinary divide in implementation nomenclature. Included studies from the present review often examined “knowledge translation interventions” or “knowledge translation strategies” (e.g., [64], [50]), and no studies specifically referenced the ERIC taxonomy or IOF. Across the rehabilitation field, the term “knowledge translation” is commonly used as a synonym for moving research into practice and is a term that has been widely accepted in the rehabilitation field since 2000 [24, 80, 81]. While international rehabilitation leaders have articulated distinctions between “knowledge translation” and “implementation science,” there is still tremendous work to be done in disseminating these distinctions to the broader rehabilitation audience [80, 81].

Further, additional research is also needed to evaluate the *cost* of implementing particular interventions in practice. Cost was the only implementation outcome that was not evaluated in any of the studies included in this review and points to a major knowledge gap in both the implementation science and stroke rehabilitation fields. Given that the lack of funds to cover implementation costs is a substantial barrier to EBP implementation in stroke rehabilitation [22], we must understand the costs associated with evidence-based interventions, programs, and assessments and the costs of using implementation strategies in stroke rehabilitation settings. One option for assessing these costs is the conduction of economic evaluations. For instance, Howard-Wilsher et al. [82] published a systematic overview of economic evaluations of health-related rehabilitation, including occupational therapy. Economic evaluations may be defined as comparing two or more interventions and examining both the costs and consequences of the intervention alternatives [82, 83]. Economic evaluations most commonly consist of cost-effectiveness analysis (CEA) but can consist of cost-utility, cost-benefit, cost-minimization, or cost-identification analysis [84, 85]. Consideration of resource allocation and costs is critically needed to make clinical and policy decisions about occupational therapy interventions [82] and should be a focus of future implementation work in occupational therapy and rehabilitation.

### Limitations

While the present scoping review adds novel contributions to the implementation science field, stroke rehabilitation, and occupational therapy, it includes several limitations. First, scoping review methodologies have been critiqued for not requiring quality and bias assessments of included articles [41, 86]. Given that this review’s focus was to synthesize the breadth of implementation strategies and outcomes measured in a field (e.g., occupational therapy) newer to implementation science, critical appraisals and bias assessments were deemed “not applicable” by the review team, a distinction that is supported by current PRISMA-ScR reporting guidelines. Second, while a comprehensive search was conducted to capture all relevant literature, the review team could have further enhanced their search strategy by consulting with an institutional librarian or performing backward/forward searching to maximize search specificity. Third, the search was restricted to studies that included occupational therapy as the primary service provider of interest. Thus, most of the studies utilized implementation strategies at the provider level. The authors recognize that the effective implementation of best practices often requires organizational- and system-level changes; therefore, the findings do not represent strategies and outcomes applicable to stroke rehabilitation clinics and the more extensive healthcare system. Lastly, the results of this scoping review returned a relatively small sample size, and therefore, conclusions should be interpreted in consideration of the available evidence.

## Conclusion

This scoping review revealed the occupational therapy profession’s use of implementation strategies and measurement of implementation outcomes in stroke rehabilitation. The fields of occupational therapy and stroke rehabilitation have begun to create a small body of implementation science literature; however, occupational therapy researchers and practitioners must continue to develop and test implementation strategies to move evidence into practice. Moreover, implementation strategies and outcomes should be described using uniform language that allows for comparisons across studies. The application of this uniform language—such as the language in the ERIC and IOF—will streamline the synthesis of knowledge (e.g., systematic reviews, meta-analyses) that will point researchers and practitioners to effective strategies that promote the use of evidence in practice. Without consistent nomenclature, it may continue to prove challenging to understand the key components of implementation strategies that are linked to improved implementation outcomes and ultimately improved care. By applying the ERIC taxonomy and IOF and using TMFs to guide study activities, occupational therapy and stroke rehabilitation researchers can advance both the fields of rehabilitation and implementation science.

## Supplementary Information

**Additional file 1.** Preferred Reporting Items for Systematic reviews and Meta-Analyses extension for Scoping Reviews (PRISMA-ScR) Checklist. The checklist contains 20 essential reporting items and two optional items when completing a scoping review [38].

**Additional file 2.** Supplemental Data File. The spreadsheet contains bibliographic search, taxonomies that guided data extraction, and the data extracted for this review.

## Data Availability

Data analyzed in this review is available in the supplemental file. The review protocol and data collection forms are available by request to the corresponding author.

## Appendix

**Table 5:** Complete electronic search strategy for PubMed (including MEDLINE) database

No.	Query	Filter	Search detail
1	(("knowledge translation" OR "research utilization") AND "occupational therap*" AND stroke)	Journal Article, English, Adult: 19+ years	("knowledge translation"[All Fields] OR "research utilization"[All Fields]) AND "occupational therap*"[All Fields] AND ("stroke"[MeSH Terms] OR "stroke"[All Fields] OR "strokes"[All Fields] OR "stroke s"[All Fields])
2	(("occupational therap*") AND ("evidence-based practice")) AND (implement*)	Journal Article, English, Adult: 19+ years	"occupational therap*"[All Fields] AND "evidence-based practice"[All Fields] AND "implement*"[All Fields]
3	(("Diffusion of Innovation"[Mesh] OR "Health Plan Implementation"[Mesh] OR "Organizational Innovation"[Mesh] OR knowledge[Tiab] OR guideline*[Tiab] OR evidence[Tiab] OR research[Tiab]) AND (implement*[Tiab] OR utiliz*[Tiab] OR diffus*[Tiab] OR translat*[Tiab] OR utilis*[Tiab])) OR ((Dissemination[Tiab] OR Diffusion[Tiab]) AND Innovation[Tiab]) OR ((increase*[tiab] OR program*[tiab] OR strateg*[tiab] OR plan*[tiab]) AND implement*[tiab]) AND ("Stroke"[Mesh] OR Cerebral-Vascular-Accident* OR Cerebrovascular-Accident* OR Stroke* OR Brain-Vascular-Accident* OR Apoplexy) AND ("Occupational Therapy"[Mesh] OR "Occupational Therapists"[Mesh] OR occupational-therap*)	Journal Article, English	((("Diffusion of Innovation"[MeSH Terms] OR "Health Plan Implementation"[MeSH Terms] OR "Organizational Innovation"[MeSH Terms] OR "knowledge"[Title/Abstract] OR "guideline*"[Title/Abstract] OR "evidence"[Title/Abstract] OR "research"[Title/Abstract]) AND ("implement*"[Title/Abstract] OR "utiliz*"[Title/Abstract] OR "diffus*"[Title/Abstract] OR "translat*"[Title/Abstract] OR "utilis*"[Title/Abstract])) OR (("Dissemination"[Title/Abstract] OR "Diffusion"[Title/Abstract]) AND "Innovation"[Title/Abstract]) OR (("increase*"[Title/Abstract] OR "program*"[Title/Abstract] OR "strateg*"[Title/Abstract] OR "plan*"[Title/Abstract]) AND "implement*"[Title/Abstract])) AND ("Stroke"[MeSH Terms] OR "cerebral vascular accident*"[All Fields] OR "cerebrovascular accident*"[All Fields] OR "stroke*"[All Fields] OR "brain vascular accident*"[All Fields] OR ("apoplexies"[All Fields] OR "Stroke"[MeSH Terms] OR "Stroke"[All Fields] OR "apoplexy"[All Fields])) AND ("Occupational Therapy"[MeSH Terms] OR "Occupational Therapists"[MeSH Terms] OR "occupational therap*"[All Fields])

The complete bibliographic search for this review is contained in the additional supplemental file for this review

## Abbreviations

OT: Occupational therapy
CFIR: Consolidated Framework for Implementation Research
ERIC: Expert Recommendations for Implementing Change
IOF: Implementation Outcomes Framework
CMS: Centers of Medicare and Medicaid Services
PRISMA-ScR: Preferred Reporting Items for Systematic Reviews and Meta-Analyses Scoping Review
CO-OP: Cognitive Orientation to daily Occupational Performance
TMF: Theory, model, or framework
RE-AIM: Reach, Efficacy, Adoption, Implementation, and Maintenance
PRISM: Practical, Robust Implementation and Sustainability Model
iPARIHS: Integrated Promoting Action on Research Implementation in Health Services
EPIS: Exploration, Adoption/Preparation, Implementation, Sustainment
TIDieR: Template for Intervention Description and Replication
SQUIRE: Standards for Quality Improvement Reporting Excellence
STaRI: Standards for Reporting Implementation studies
JBI: Joanna Briggs Institute
